# Antimicrobial Resistance and Genomic Characterization of *Campylobacter jejuni* and *Campylobacter coli* Isolated from Retail Chickens in Beijing, China

**DOI:** 10.3390/microorganisms12081601

**Published:** 2024-08-06

**Authors:** Yao Bai, Jiaqi Ma, Fengqin Li, Baowei Yang, Xiu Ren, Yeru Wang, Yujie Hu, Yinping Dong, Wei Wang, Jing Zhang, Shaofei Yan, Shenghui Cui

**Affiliations:** 1 NHC Key Laboratory of Food Safety Risk Assessment, China National Centre for Food Safety Risk Assessment, Beijing 100022, China; baiyao@cfsa.net.cn (Y.B.);; 2 College of Food Science and Engineering, Northwest Agriculture and Forestry Science and Technology University, Shaanxi 712100, China; mjq1531900880@nwafu.edu.cn (J.M.);; 3 National Institutes for Food and Drug Control, Beijing 100050, China

**Keywords:** *Campylobacter jejuni*, *Campylobacter coli*, antimicrobial resistance, whole genome sequencing

## Abstract

Objective *Campylobacter* species are the main causes of foodborne illness worldwide, posing significant threats to public health. This study aimed to investigate the antibiotic resistance and genomic characterization of *C. jejuni*/*C.coli* from retail chickens in Beijing. Methods Antimicrobial susceptibility testing was conducted on 126 *C. jejuni*/*C. coli* isolated from retail chickens in Beijing, following CLSI protocols. Whole genomes of all isolates were sequenced using the Illumina platform. Results More *C. coli* (83.82%) showed multi-drug resistance than *C. jejuni* (8.62%). Genomic analysis demonstrated 42 sequence types (STs) and 12 clonal complexes (CCs), from which CC828 and CC52 were dominant. *cdtA*, *cdtB* and *cdtC* encoding cytotoxic protein were present spontaneously in most *C. jejuni* but not found in any *C. coli* isolates. The abundances of antibiotic resistance genes (ARGs) and virulence genes (VGs) in *C. jejuni* and *C. coli* were significantly different, with ARGs numbered in *C. coli* and VGs in *C. jejuni*. Conclusions High prevalence of multi-drug resistance *C. coli* and *C. jejuni* isolated from Beijing chickens were challenging clinical antibiotic usages in the treatment of *Campylobacter* infection. The surveillance of particular *C. jejuni* and *C. coli* STs correlated with higher resistance and virulence needs to be strengthened in the future.

## 1. Introduction

*Campylobacter* continues to be recognized as one of the major zoonotic pathogens causing foodborne diarrheal illnesses. Approximately 500 million cases of gastrointestinal infections caused by *Campylobacter* have been reported globally [1]. Currently, 61 species and 16 subspecies belonging to the genus *Campylobacter* have been identified (http://www.bacterio.net/, accessed on 27 May 2024). Among these species, *Campylobacter jejuni* and *Campylobacter coli* altogether are responsible for over 90% of all human *Campylobacter* gastroenteritis cases, of which approximately 30% of acute enteritis caused by *C. jejuni* develop severe irritable bowel syndrome with a disease fatality rate about 5/100,000 [1]. Poultry, especially chickens, were the primary reservoirs for *Campylobacter* spp., mainly *C. jejuni* and *C. coli* [2]. If chicken products are contaminated with *Campylobacter* during food processing or sales, humans may become infected after consuming these contaminated chicken products [3].

*Campylobacter* infection is a self-limiting process that typically does not require antibiotic treatment [4]. However, for patients suffering severe symptoms from infection or with compromised immune systems, treatment with antibiotics, such as erythromycin, tetracyclines, aminoglycosides and fluoroquinolones, is necessary. Fluoroquinolones, especially ciprofloxacin, have long been considered the main antibiotics for the treatment of Campylobacteriosis [5]. As antimicrobial resistance in *Campylobacter* becomes more severe in many countries, especially with the emergence of multi-drug resistance (MDR), significant concerns regarding food safety and public health have gained attention internationally [6]. Furthermore, antibiotic resistance genes (ARGs) have been identified in *Campylobacter* isolates from various sources, especially chicken meats and their related products. Epidemiological research has further confirmed that the prevalence of *Campylobacter* resistant to clinically relevant antibiotics, such as fluoroquinolones, macrolides and aminoglycoside, has increased remarkably during recent years [7].

Recent studies have identified the major virulence factors involved in the pathogenesis of *Campylobacter* isolates. *Campylobacter* can adhere to host cells through the expression of *flaA*, *cadF*, *jlpA*, *porA* and *dnaJ* genes, invade intestinal epithelial cells through the expression of *ciaB* and *ceuE* genes and produce toxins and survive in host cells through the expression of *cdtA*, *cdtB* and *cdtC* genes. In addition, studies have found a correlation between *Campylobacter* virulence genes and antibiotic resistance, which indicates a link between antibiotic resistance and the colonization or invasion ability of these bacteria [8].

Since antibiotic resistance and virulence status of *Campylobacter* isolates could be diversified in various ways depending on the antibiotic usage or investigation period and region, it is necessary to assess the antibiotic resistance and virulence distributing patterns through surveillance. To date, many countries are continuously investigating antibiotic resistance and virulence against pathogenic bacteria at the national level [9]. In 2019, a gastroenteritis outbreak caused by MDR *C. coli* was identified in Beijing, China, indicating the importance of *Campylobacter* surveillance to protect human health [10]. In food contamination surveillance in China, *C. jejuni* and *C. coli* were mainly isolated from food-producing animals, especially chickens [11]. However, to the best of our knowledge, the genetic traits of the coexistence of antibiotic-resistant genes and virulence genes of *Campylobacter* in retail chickens in Beijing, China, need to be further elucidated.

The purpose of this study was to investigate the antimicrobial susceptibility and assess the genomic variation of *C. jejuni* and *C. coli* isolated from retail chickens in Beijing.

## 2. Materials and Methods

### 2.1. Campylobacter Isolates Collection and Preparation

In total, 126 isolates of *Campylobacter* isolated from retail chickens in Beijing, China, 2018–2020, were used. All isolates were stored at −80 °C in Brucella broth supplemented with 50% glycerol (*v*/*v*) and 5% laked sheep blood (*v*/*v*). The isolates were revived in Bolton broth, incubated at 42 °C for 48 h under microaerophilic conditions (5% O_2_, 10% CO_2_ and 85% N_2_), streaked on Columbia blood agar plate and incubated at 42 °C for 24 h under microaerophilic conditions (5% O_2_, 10% CO_2_ and 85% N_2_). All isolates were identified by PCR assay [12] and confirmed by Vitek 2 with NH card.

### 2.2. Antimicrobial Susceptibility Test

The antimicrobial susceptibility of the *C. jejuni* (*n* = 58) and *C. coli* (*n* = 68) isolates were tested and interpreted using the broth microdilution method, according to the guidelines of the Clinical and Laboratory Standard Institute (CLSI, M45-A3) and EUCAST epidemiological cut-off values (ECOFFs), as shown in Supplementary Table S6. Minimal inhibitory concentrations (MICs) to 6 antimicrobials were determined via broth microdilution, including chloramphenicol (CHL), ciprofloxacin (CIP), erythromycin (ERY), doxycycline (DC), gentamicin (GEN) and tetracycline (TET). Reference strain *C. jejuni* ATCC 33560 was used as quality control for antimicrobial susceptibility experiments. The isolates that showed resistance to three or more antibiotic categories are defined as multi-drug resistant (MDR) [13,14], while in this study, only antibiotics with CLSI breakpoints were taken into account during the calculation of MDR.

### 2.3. Whole Genome Sequencing

Genomic DNA (gDNA) was extracted using Omega EZNA Bacterial DNA Kit (Omega Bio-tek, Norcross, GA, USA). The gDNA was sent to Novogene for whole genome sequencing, where Illumina HiSeq protocol was used on an Illumina PE150 platform; SOAPdenovo v2.04 (https://github.com/aquaskyline/SOAPdenovo2, accessed on 27 May 2024), SPAdes v3.15.4 (https://github.com/ablab/spades, accessed on 27 May 2024) and AbySS v2.1.5 (https://github.com/bcgsc/abyss, accessed on 27 May 2024) were used for the initial assembly process. Then, GapCloser v1.12 (https://anaconda.org/bioconda/soapdenovo2-gapcloser, accessed on 27 May 2024) was used to refine the assembly. The species identification was reconfirmed using the SpeciesFinder 2.0 on the Centre for Genomic Epidemiology (CGE) website (https://www.genomicepidemiology.org, accessed on 27 May 2024).

### 2.4. Screening of Antimicrobial Resistance Genes (ARGs), Virulence Genes and Point Mutations

ARGs and virulence genes of *Campylobacter* genomes were annotated via Resfinder, Virulence Factor Database (VFDB) databases (updated to 27 May 2024) through ABRicate v1.01 (https://github.com/tseemann/abricate, accessed on 27 May 2024). The thresholds for identity and coverage were all set to 80 percent. Point mutations were predicted by Pointfinder 3.1.0 (https://bitbucket.org/genomicepidemiology/pointfinder, accessed on 27 May 2024) and confirmed by comparing with reference genome NCTC 11168 (GCF_000009085.1).

### 2.5. Multilocus Sequence Typing

In silico multilocus sequence typing (MLST) was conducted using the MLST database on the public databases for molecular typing and microbial genome diversity (PubMLST) website (https://pubmlst.org/, accessed on 27 May 2024). New alleles and sequence types (STs) were submitted to the PubMLST database. A minimum spanning tree of *Campylobacter* isolates was generated on the basis of the MLST results using PHYLOVIZ Online (https://online.phyloviz.net/index, accessed on 27 May 2024).

### 2.6. Core Genome Phylogenetic Analysis

The chromosomes were annotated using the prokaryotic genome annotation tool Prokka (v1.12). Core genomes of all assemblies were determined using Roary (v3.11.0) and aligned using MAFFT (v7.313). A maximum-likelihood phylogenetic tree of the aligned genomes was constructed using FastTree (v2.1.10). The phylogenetic relationship of *Campylobacter* isolates was visualized using the ChiPlot tool (https://www.chiplot.online/, accessed on 27 May 2024) [15].

### 2.7. Statistical Analysis

Statistical analysis of the data was performed using R version 4.2.2 (http://www.r-project.org/, accessed on 27 May 2024) and IBM SPSS 26 (IBM SPSS, Armonk, NY, USA). The distribution of *Campylobacter* was visualized by the Sankey plot. Differences in the total number of ARGs and VFs among different species and sources of *Campylobacter* were assessed by the Mann–Whitney–Wilcoxon test. Among different *Campylobacter*, cluster heatmap analyses on the prevalence of antibiotic resistance (AMR), specific ARGs and VFs were performed, respectively.

The association between the resistance profile of each antimicrobial and the presence/absence of ARGs, point mutations and virulence genes was assessed using binary logistic regression models. AMR was considered as a binary dependent variable (0 = non-resistant; 1 = resistant).

The correlation between the resistance profile of each antimicrobial and the presence/absence of ARGs and point mutations was analyzed separately for *C. coli* and *C. jejuni*. The ARGs *bla*_OXA-184_, *bla*_OXA-185_ and *bla*_OXA-465_ and point mutations L4 (V82I, T91K, V176I, T177S) and, GyrA (S22G, T86I) were excluded from *C. coli* correlation analysis for either 100% presence or 100% absence in *C. coli*. The ARGs *aac(6′)-aph(2″)*, *aadE-Cc*, *erm*(B), *fexA*, *tet*(L) and *bla*_OXA-489_ and point mutation 23S (A2075G) were excluded from *C. jejuni* correlation analysis for 100% absence in *C. jejuni*. The antibiotics ciprofloxacin (100% resistance) was excluded from correlation analysis *of C. coli*.

The evaluation of the correlation between the resistance profile of each antimicrobial and the presence/absence of virulence genes was also carried out separately for *C. coli* and *C. jejuni*. The VFs *cadF*, *pebA*, *jlpA*, *porA*, *kpsD*, *kpsF*, *neuA1*, *wlaN*, *ciaB*, *ciaC*, *flgB*, *flhB*, *cdtA*, *cdtB* and *cdtC* were excluded from *C. coli* correlation analysis for either 100% presence or 100% absence in *C. coli*. The VFs *cadF*, *pebA*, *jlpA*, *cheA*, *htrB*, *kpsD*, *kpsF*, *ciaB*, *ciaC*, *flgB* and *flhB* genes were excluded from *C. jejuni* correlation analysis for 100% presence in *C. jejuni*. The antibiotic ciprofloxacin (100% resistance) was excluded from the correlation analysis *of C. coli*. The differences between variables were considered statistically significant when *p* value < 0.05.

## 3. Results

### 3.1. Antimicrobial Susceptibility Test

**Figure 1:**
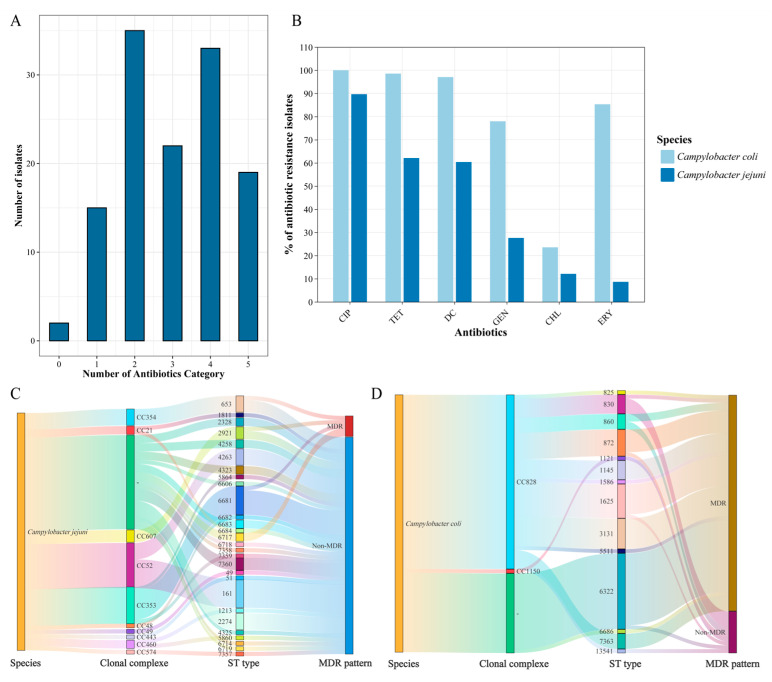
Antimicrobial susceptibility characterization of *Campylobacter* isolates from retail chickens. (**A**) Percentages and numbers of MDR *Campylobacter* isolates resistant to various antibiotics; (**B**) Percentage of *C. coli* and *C. jejuni* isolates resistant to various antibiotics; (**C**) Distribution of *C. jejuni* isolated from retail chickens in Beijing, China; (**D**) Distribution of *C*. *coli* isolated from retail chickens in Beijing, China. The line indicates the distribution of the *Campylobacter* in species, MDR pattern, clonal complexes and sequence types (STs).

Overall, 124 *Campylobacter* isolates were resistant to at least one antibiotic. Seventy-four (58.73%) isolates were MDR (Figure 1A). Resistance to ciprofloxacin was detected in *C. coli* (100%) and *C. jejuni* (89.66) isolates.

For *C. coli* isolates, resistance to tetracycline accounted for a large proportion (98.53%), followed by resistance to doxycycline (97.06%), erythromycin (85.29%), gentamicin (77.94%) and chloramphenicol (23.53%). For *C. jejuni*, similarly, resistance to tetracycline also accounted for a large proportion (62.07%), followed by resistance to doxycycline (60.34%), gentamicin (27.59%), chloramphenicol (12.07%) and erythromycin (8.62%) (Figure 1B).

The resistance rates of *C. jejuni* isolates against each of the six antibiotics were all lower than those of *C. coli* isolates. More than half of *C. coli* (83.82%) and *C. jejuni* (8.62%) were found to exhibit MDR. Sankey plot showed a widespread prevalence and high diversity of *Campylobacter* during distribution analysis of sampling locations, sample types and MDR pattern (Figure 1C,D).

### 3.2. Sequence Types and Clonal Complexes

**Figure 2:**
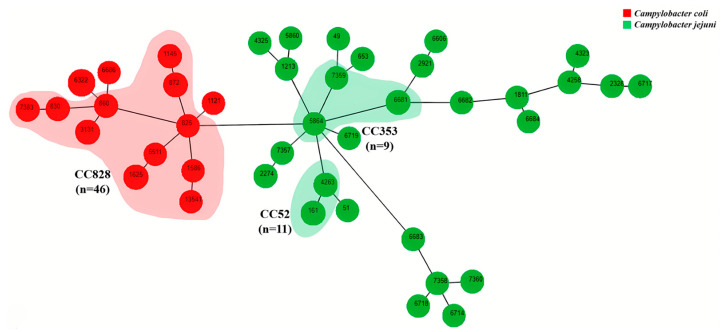
Minimum spanning tree of *Campylobacter* isolates. Each node represents one ST. The size of the node is related to the number of isolates. Branch length between nodes indicates genetic distance based on the nucleotide differences among seven housekeeping genes of *Campylobacter*. The colors of nodes represent *Campylobacter* species: red, *C. coli*; green, *C. jejuni*. Main CCs are shown in the shaded area.

In total, 42 STs and 13 clonal complexes (CCs) were detected in 126 *Campylobacter* isolates (Figure 1C and Figure 2).

In *C. coli,* the most common CC was CC828 *C. coli* (*n* = 46; 67.65%), which consisted of 11 STs: ST825, ST830, ST860, ST872, ST1145, ST1586, ST1625, ST3131, ST5511, ST7363 and ST13541. The most frequent CC in *C. jejuni* was CC52 (*n* = 11; 18.97%), which comprised ST161 and ST4263. The remaining CCs included relatively fewer STs or a small number of strains. The *C. jejuni* isolates from chickens (*n* = 58) showed 28 STs; among these, ST161 (*n* = 7; 12.07%) and ST6681 (*n* = 7; 12.07%) were the most prevalent. The *C. coli* isolates from chickens (*n* = 68) showed 14 STs, among which ST6322 (*n* = 20; 29.41%), ST1625 (*n* = 9; 13.24%) and ST3131 (*n* = 8; 11.76%) were the most common. In addition, after verification by PubMLST, a new ST13541 of *C. coli* was identified in this study. No *C. coli* isolates from Clade 2 or 3 were found in this study.

### 3.3. Analysis of Antibiotic Resistance Genes and Point Mutations

**Figure 3:**
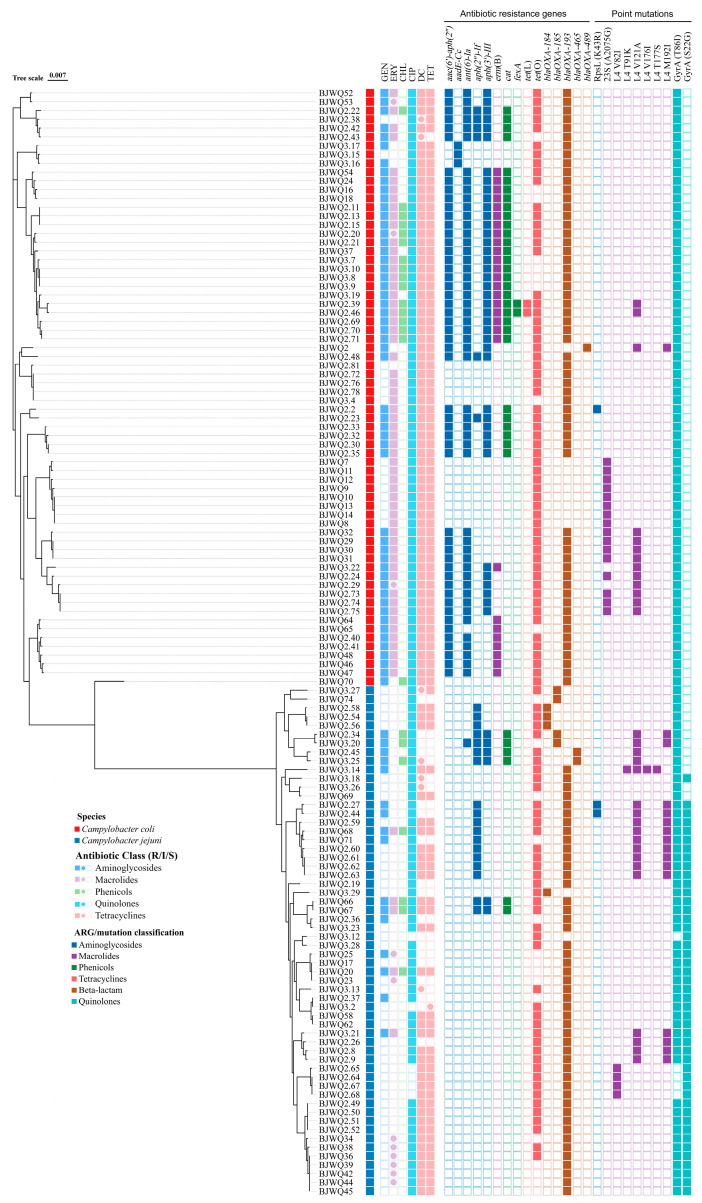
Phylogenetic analysis and resistance heatmap of 126 *Campylobacter* isolates. Different colors were used to indicate the species, antibiotic phenotypes, ARGs and mutation-related resistance mechanisms. The presence and absence of ARGs and point mutations are denoted by filled and hollow squares, respectively.

A total of 15 different ARGs and 10 point mutations encoding resistance to six antimicrobial classes were detected in 126 isolates, including β-lactam (*n* = 5), aminoglycoside (*n* = 6), quinolone (*n* = 2), phenicol (*n* = 2), tetracycline (*n* = 2) and macrolide (*n* = 8) (Figure 3). The gene encoding β-lactam resistance was observed for *bla*_OXA-__193_ (84.13%), followed by *tet*(O) (80.95%) encoding tetracycline resistance. Detection rates of the remaining ARGs were <50%.

High prevalence of mutation T86I in protein GyrA was found in 96.03% (121/126) of all the *Campylobacter* isolates, while S22G was found in only 36.51% (46/126) of them. One mutation of RpsL (K43R) and six mutations (V82I, T91K, V121A, V176I, T177S and M192I) on ribosomal protein L4 was found, from which V121A and M192I were present in 31 and 16 isolates. Mutation on the 23S rRna gene (A2075G) was found in 16 isolates.

In *C. coli*, high prevalence rates of GyrA (T86I) (100%), *bla*_OXA-__193_ (86.76%) encoding β-lactam resistance and *tet*(O) (86.76%) and *aac(6′)-aph(2″)* (75%), *ant(6)-Ia* (73.53%) and *aph(3′)-III* (58.82%) encoding aminoglycoside resistance were observed.

In *C. jejuni*, the most frequent one was GyrA (T86I) (91.38%), followed by *bla*_OXA-193_ (81.03%) encoding β-lactam resistance and GyrA (S22G) (79.31%) and *tet*(O) (74.14%) encoding tetracycline resistance (Figure 3). The average number of ARGs in *C. coli* was significantly higher than that in *C. jejuni* (Figure 3). The genes *aac(6′)-aph(2″)* (75%), *erm*(B) (41.18%), 23S (A2075G) (23.53%), *aadE-Cc* (4.41%), *fexA* (2.94%), *tet*(L) (2.94%) and *bla*_OXA-489_ (1.47%) were detected only in *C. coli*, while GyrA (S22G) (79.31%), L4 V82I (6.89%), *bla*_OXA-184_ (6.90%), *bla*_OXA-185_ (6.90%), *bla*_OXA-465_ (3.45%), L4 T177S (1.72%), L4 V176I (1.72%) and L4 T91K (1.72%) were detected only in *C. jejuni* (Figure 3).

### 3.4. Analysis of Virulence Genes

**Figure 4:**
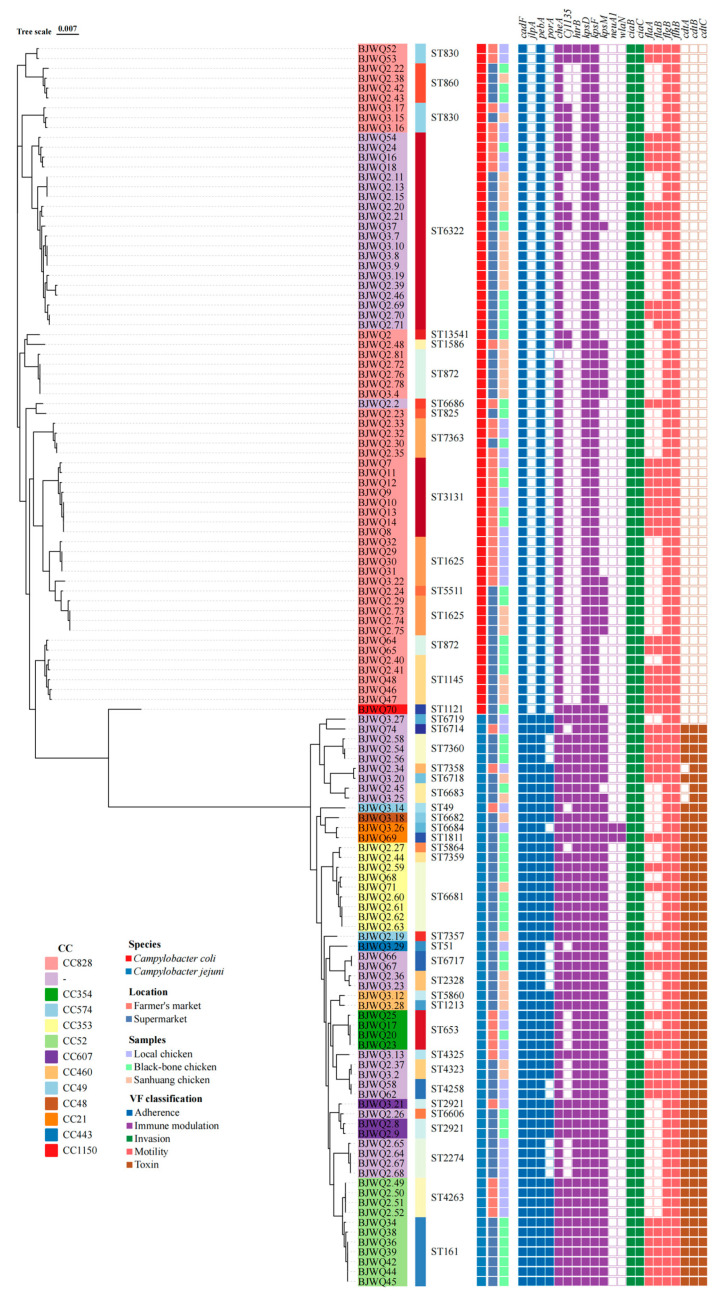
Phylogenetic analysis and virulence heatmap of 126 *Campylobacter* isolates. Different colors were used to indicate locations, sample types, STs, CCs, species and VF classifications. Unassigned CCs are denoted by short lines (-). The presence and absence of VGs are denoted by filled and hollow squares, respectively.

In total, 21 virulence genes classified into five VF classes, such as adherence, immune modulation, invasion, motility and toxin, were identified in 126 *Campylobacter* isolates (Figure 4). High prevalence rates were observed for the virulence genes *cadF* (100%) and *pebA* (100 %) encoding adherence; *kpsD* (100%), *kpsF* (100%), *cheA* (99.21%) and *kpsM* (56.35%) encoding immune modulation; *ciaB* (100%) and *ciaC* (100%) encoding invasion; and *flgB* (100%) and *flhB* (100%) encoding motility (Figure 4). Detection rates of the remaining virulence genes were less than 50 %. Type VI secretion system was absent in all isolates.

This study also investigated the effects of various factors on the number of virulence genes carried by the *Campylobacter* isolates. Similar to ARGs, the mean number of virulence genes in *Campylobacter* was found to be significantly higher in isolates from supermarkets than those from farmers’ markets (*p* < 0.05), whereas the mean number of virulence genes in *Campylobacter* isolates from black-bone chickens was significantly higher than that in isolates from local chickens.

**Figure 5:**
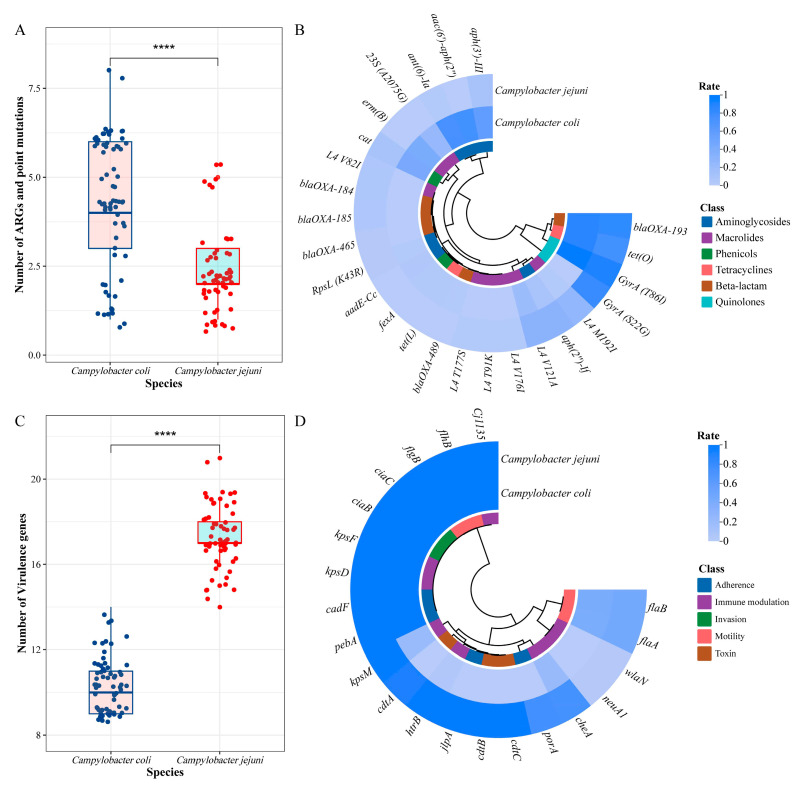
Statistical analysis of ARGs, point mutations and VGs in *Campylobacter* isolates from retail chickens. (**A**) Total number of ARGs and mutation classifications from *C. coli* and *C. jejuni*; (**B**) Prevalence of ARGs and mutation classifications; (**C**) Total number of VGs from *C. coli* and *C. jejuni*; (**D**) Prevalence of VGs. *p* value is based on the Wilcoxon signed-rank test (**** *p* < 0.0001).

All *C. coli* and *C. jejuni* harbored the virulence genes *cadF*, *pebA*, *kpsD*, *kpsF*, *ciaB, ciaC*, *flgB* and *flhB* correlated with adherence, immune modulation, invasion and motility functions. The average number of virulence genes in *C. jejuni* was significantly higher than that in *C. coli* (Figure 5C). Notably, *C. jejuni* harbored all three *cdt* genes at high prevalence levels, such as *cdtA* (93.10%), *cdtB* (98.27%) and *cdtC* (98.27%), while *cdtA*, *cdtB* and *cdtC* genes were not found in all *C. coli* isolates (Figure 4).

### 3.5. Phylogenetic Analysis

To investigate the genetic relationship among *Campylobacter* isolates from retail chickens in Beijing, a maximum likelihood tree was constructed using core single nucleotide polymorphisms (SNPs) identified in the 126 *Campylobacter* isolates (Figure 4). As shown in the phylogenetic tree, the 126 *Campylobacter* strains clustered distinctly into two branches: *C. coli* and *C. jejuni*.

The 68 *C. coli* isolates belonged to CC828, CC1150 and an unassigned CC, whereas the 58 *C. jejuni* isolates belonged to 10 distinct CCs (CC21, CC48, CC49, CC52, CC57, CC607, CC353, CC354, CC443 and CC46) and an unassigned CC.

In *C. coli*, seven isolates from ST830 and ST872 displayed the least resistance genes and were sensitive to most antimicrobial agents. Seventeen isolates from ST860, ST1145, ST6332 and ST7363 harbored the most resistance genes and were resistant to most antimicrobial agents. Although ARGs were comparatively less prevalent in *C. jejuni* than in *C. coli*, it is intriguing that seven isolates of ST653, ST6717, ST6681, ST6683 and ST7360 carry more resistance genes than other *C. coli* (Figure 3 and Figure 4).

### 3.6. Association between Antibiotic Resistance and Presence of ARGs and Point Mutations

Binary logistic regression models were applied for each *Campylobacter* isolate, using the antimicrobial resistance profiles as a dependent variable and the presence/absence of ARGs and point mutations as independent variables. It was observed that *C. coli* and *C. jejuni* showed a statistically significant association (*p* < 0.05) between antimicrobial resistance and the presence of ARGs. In *C. coli* isolates, the *tet*(O) gene showed a positive association with chloramphenicol (Figure 5 and Supplementary Table S1). In *C. jejuni* isolates, the *aph(2″)-If* gene showed a positive association with erythromycin resistance, the *tet*(O) gene showed a positive association with erythromycin resistance, and the L4 M192I gene showed a positive association with gentamicin and chloramphenicol resistance (Figure 5 and Supplementary Table S2).

### 3.7. Association between Antibiotic Resistance and Presence of Virulence Genes

Binary logistic regression models were applied for each *Campylobacter* isolate, using the antimicrobial resistance profiles as a dependent variable and the presence/absence of virulence genes as an independent variable. It was observed that *C. coli* and *C. jejuni* showed a statistically significant association (*p* < 0.05) between antimicrobial resistance and the presence of virulence genes. In *C. coli* isolates, the *Cj1135* gene showed a negative association with resistance to doxycycline; the *kpsM* gene showed a negative association with resistance to gentamicin, erythromycin and chloramphenicol (Figure 5 and Supplementary Table S3). In *C. jejuni* isolates, the *Cj1135* gene showed a positive association with resistance to gentamicin, erythromycin, ciprofloxacin and tetracycline (Figure 5 and Supplementary Table S4).

## 4. Discussion

*Campylobacter*, one of the four main causes of gastroenteritis worldwide, poses a serious threat to food safety and public health. At present, the contamination of MDR *Campylobacter* isolates in food products and their hypervirulent potential have globally raised public safety concerns. However, there is limited research in China on the antibiotic resistance phenotypes, ARGs and virulence genes of *Campylobacter* isolated from chickens sold in supermarkets and farmers’ markets. In this study, we first evaluated the antibiotic resistance phenotype and MDR of *Campylobacter* in retail chickens from supermarkets and farmers’ markets in Beijing and revealed the molecular characteristics of *Campylobacter* isolates. Genomic analysis of pathogens in retail chickens provides a more comprehensive and better understanding of the characteristics of *Campylobacter* isolated from chickens, which may be used to inform prevention strategies. Nevertheless, few studies have been conducted in China to examine the genomic characteristics of *Campylobacter* in retail chickens from supermarkets. The genome sequences of *Campylobacter* obtained in this study can provide a source of information on pathogens in retail chickens for researchers worldwide.

Owing to the extensive use of antibiotics in human health care, livestock production and agriculture, antibiotic resistance has emerged as a major global concern [16]. Of all the 126 *Campylobacter* isolates in our study, 49.21% (62/126) were MDR, and 15.08% (19/126) were resistant to antibiotics of at least five antimicrobial classes. The highest resistance to ciprofloxacin, tetracycline and doxycycline was observed in this study, which was consistent with previous reports [17]. The proportion of MDR isolates in this study is markedly higher than that reported in Japan, Spain and Canada [18,19,20]. The resistance of *C. coli* was more severe than that of *C. jejuni*, especially ST6322 *C. coli* isolates, which were 95% (19/20) MDR. However, it is worth noting that the antibiotic resistance rate of *C. jejuni* isolated from diarrhea patients in Beijing is generally lower than that of this study [21]. Remarkably, four *C. jejuni* isolates showed resistance to six antibiotics, which indicates that strengthened surveillance of the AMR status in *Campylobacter* needs to be implemented in the future.

For ARGs, we found that a high percentage of *C. coli* (32.35%) and *C. jejuni* (77.59%) strains carried *tet*(O), which is consistent with previous reports, indicating that *Campylobacter* exhibits high levels of tetracycline resistance [22]. According to reports, the *tet*(O) gene is the only tetracycline resistance determinant found in *Campylobacter* and is widely detected in all tetracycline-resistant *Campylobacter* isolates [22]. The mutation T86I in protein GyrA was associated with quinolone resistance in *Campylobacter* [23]. All isolates showed resistance to ciprofloxacin, and the high prevalence of GyrA (T86I) (96.03%, 121/126) in all *Campylobacter* isolates explained resistance to ciprofloxacin in 121 isolates. The other five isolates resistant to ciprofloxacin without T86I were found. Another mutation (S22G) out of the QRDR of GyrA, which correlated with quinolone resistance [24], was found in another five isolates resistant to ciprofloxacin but without T86I mutation. The high prevalence of the *tet*(O) gene in our isolates indicates that the *Campylobacter* isolates from Beijing chickens have high tetracycline resistance. Among aminoglycoside resistance genes, *aadE-Cc*, *aph(3′)-III*, *aac(6′)-aph(2″)* and *ant(6)-Ia* were observed to be more common in *C. coli* than in *C. jejuni* in the present study, correlating well with resistance phenotypes of *C. coli* and *C. jejuni*, which is consistent with previous findings [22]. The *cat* gene and *fexA* gene encode resistance to phenicols, whereas the *erm*(B) gene encodes resistance to macrolides. Remarkably, although the *cat* (10.34%) gene was detected at a lower frequency in *C. jejuni*, and the *fexA* gene and *erm*(B) gene were not detected, *C. jejuni* still exhibited resistance to chloramphenicol (12.07%) and erythromycin (8.62%). It has been reported that mutations on RpsL, ribosomal protein L4 and 23S rRNA gene were responsible for macrolides resistance of *Campylobacter* [25]. In our study, we discovered RpsL (K43R) and six mutations (V82I, T91K, V121A, V176I, T177S and M192I) on ribosomal protein L4, and the 23S rRNA gene (A2075G) was found mostly in *cat-, erm*(B)- and *fexA*-absent *C. jejuni* isolates, partially implicating the mechanism of macrolides resistance of *C. jejuni*.

In this study, MLST typing was used to investigate the genotypes of chicken-derived *Campylobacter* from supermarkets and farmers’ markets in Beijing. Our findings demonstrated that 126 *Campylobacter* isolates were classified into 42 STs, with strains belonging to CC828 as the predominant group. CC828 is the largest and most widely distributed CC worldwide, representing 21.08% of all *Campylobacter* strains submitted to the PubMLST database. As reported by numerous studies, the major CCs of *Campylobacter* vary by country and region, but CC21 (16.39%), CC353 (7.10%), CC45 (6.12%) and CC48 (5.01%) are consistently the predominant CCs among isolates in the PubMLST database. CC828 is the most frequently isolated CC from chickens, and the majority of isolates belonging to CC828 are *C. coli* isolates [26]. In the present study, the prevalences of CC52 (*n* = 11; 8.73%) and CC353 (*n* = 9; 7.14%) were lower than that of CC828. It is worth noting that the main source of CC52 isolates is humans, but the prevalence of CC52 isolates in chickens was relatively high in this study. This result is consistent with previous findings, suggesting a potential bidirectional transmission of these strains between humans and chickens [27]. Notably, genetic evolution analysis showed that not all isolates with the same STs originated from the same sampling location and type of chicken, and some ST isolates were cross-distributed in different supermarkets and types of chicken; this finding is consistent with previously reported findings [28]. *Campylobacter* isolated from broiler chickens in eastern China mainly includes *C. jejuni* ST8089, ST10242, ST10244 and ST10243 and *C. coli* ST1121, ST830, ST1568, ST1625, ST872 and ST829 [29]. Maesaar et al. (2018) reported ST5, ST45 and ST50 as the most prevalent genotypes of *C. jejuni* isolates from poultry chickens in Estonia [30]. The prevalent STs of *C. jejuni* isolated in the present study do not correspond with those mentioned above. However, the detected *C. coli* STs ST830, ST872, ST1121, ST1568 and ST1625 are almost identical to the ones mentioned above. These data highlight the wide-range transmission of these strains across regions and indicate that ST diversity varies among countries and regions. In addition, a *C. coli* strain (BJWQ2) belonged to an ST that had not been reported previously. Sequence data from this strain were submitted to the *Campylobacter* MSLT database (PubMLST), which led to the assignation of a novel ST (ID PubMLST 13541) and a novel allele sequence for *gltA* (ID PubMLST 841).

The pathogenesis of *Campylobacter* to cause diarrhea is complicated. Previous studies showed that genes involved in epithelial cell motility, colonization, invasion and toxin production play an important role in the development of *Campylobacter*-related diseases [31]. The *flaC*, *ciaB*, *pebA* and *cadF* genes were highly prevalent in the *Campylobacter* isolates in this study; nevertheless, there are divergences regarding the constitutive presence of these genes in this microorganism [31]. The presence of the *cadF* gene is crucial for pathogenesis, as adhesion is a prerequisite process for any bacterial pathogens to invade epithelial cells; our results demonstrated ubiquitous invading potentiality in all *Campylobacter* isolates from retail chickens in this study. The invasion of epithelial cells and the production of CDT are important bacterial virulence mechanisms that play a key role in enterocolitis. While the presence of a single cdt gene has no effect on bacterial virulence, the coexistence of all three *cdt* genes leads to the production of functional cytotoxic substances [32]. In our study, *cdtA*, *cdtB* and *cdtC* were present spontaneously in most *C. jejuni* isolates but were not found in all *C. coli* isolates, indicating that *C. jejuni* is more virulent than *C. coli* in retail chickens in Beijing.

Phylogenetic analysis provides information on the prevalence and phylogenetic relationships of antibiotic resistance phenotypes, ARGs and virulence genes of *Campylobacter*. A previous study found that the virulence genes *cadF* and *ciaB* affect chloramphenicol and ampicillin resistance of *Campylobacter* [8]. Similarly, in our study, analysis of the relationship between the presence of virulence genes and antimicrobial resistance in *C. jejuni* isolates showed consistent positive (*p* < 0.05 and an OR > 1) correlations. The mechanisms underlying the correlation between antibiotic resistance and virulence genes in *Campylobacter* remains unclear to date, for which further research is needed.

## 5. Conclusions

Antimicrobial resistance of *C. coli* from retail chickens in Beijing is severe, especially with the high prevalence of MDR *C. coli* isolates. Although the antimicrobial resistance level of *C. jejuni* isolates is relatively milder than *C. coli*, their stronger invading potentiality is of great concern to public health. Therefore, it might be challenging for clinical antibiotic usage in the treatment of illnesses caused by *Campylobacter*. Meanwhile, the distinctive sequence types of *C. jejuni* and *C. coli* correlating with higher resistance and virulence genotypes in chickens need to be considered, particularly in future surveillance.

## Data Availability

Data will be made available upon request.

## Supplementary Materials

The following supporting information can be downloaded at: https://www.mdpi.com/article/10.3390/microorganisms12081601/s1, Table S1. Relationship between Susceptibility/Resistance Patterns and Associated Antibiotic Resistance Genes and Point Mutations in *C. coli* Isolates; Table S2. Relationship between Susceptibility/Resistance Patterns and Associated Antibiotic Resistance Genes and Point Mutations in *C. jejuni* Isolates; Table S3. Relationship between Susceptibility/Resistance Patterns and Associated Virulence Genes in *C. coli* Isolates; Table S4. Relationship between Susceptibility/Resistance Patterns and Associated Virulence Genes in *C. jejuni* Isolates. Table S5. Reference ARG Sequences Used in This Study. Table S6. The Interpretative Standards for Antibiotic Resistance Used in This Study.

## References

[1] Igwaran A., Okoh A.I. (2019). Human Campylobacteriosis: A public health concern of global importance. Heliyon.

[2] Taha-Abdelaziz K., Singh M., Sharif S., Sharma S., Kulkarni R.R., Alizadeh M., Yitbarek A., Helmy Y.A. (2023). Intervention strategies to control *Campylobacter* at different stages of the food chain. Microorganisms.

[3] Mota-Gutierrez J., Lis L., Lasagabaster A., Nafarrate I., Ferrocino I., Cocolin L., Rantsiou K. (2022). *Campylobacter* spp. prevalence and mitigation strategies in the broiler production chain. Food Microbiol..

[4] Ben Romdhane R., Merle R. (2021). The data behind risk analysis of *Campylobacter jejuni* and *Campylobacter coli* infections. Curr. Top. Microbiol. Immunol..

[5] Di Giannatale E., Calistri P., Di Donato G., Decastelli L., Goffredo E., Adriano D., Mancini M.E., Galleggiante A., Neri D., Antoci S. (2019). Thermotolerant *Campylobacter* spp. in chicken and bovine meat in Italy: Prevalence, level of contamination and molecular characterization of isolates. PLoS ONE.

[6] Qin X., Wang X., Shen Z. (2023). The rise of antibiotic resistance in *Campylobacter*. Curr. Opin. Gastroenterol..

[7] Mourkas E., Florez-Cuadrado D., Pascoe B., Calland J.K., Bayliss S.C., Mageiros L., Méric G., Hitchings M.D., Quesada A., Porrero C. (2019). Gene pool transmission of multidrug resistance among *Campylobacter* from livestock, sewage and human disease. Environ. Microbiol..

[8] Gharbi M., Béjaoui A., Ben Hamda C., Ghedira K., Ghram A., Maaroufi A. (2022). Distribution of virulence and antibiotic resistance genes in *Campylobacter jejuni* and *Campylobacter Coli* isolated from broiler chickens in Tunisia. J. Microbiol. Immunol. Infect..

[9] Yadav S., Kapley A. (2021). Antibiotic resistance: Global health crisis and metagenomics. Biotechnol. Rep..

[10] Li Y., Zhou G., Gao P., Gu Y., Wang H., Zhang S., Zhang Y., Wang Y., Jing H., He C. (2020). Gastroenteritis outbreak caused by *Campylobacter jejuni*—Beijing, China, August 2019. China CDC Wkly.

[11] Bai Y., Cui S., Xu X., Li F. (2014). Enumeration and characterization of *Campylobacter* species from retail chicken carcasses in Beijing, China. Foodborne Pathog. Dis..

[12] Wang G., Clark C.G., Taylor T.M., Pucknell C., Barton C., Price L., Woodward D.L., Rodgers F.G. (2002). Colony multiplex PCR assay for identification and differentiation of *Campylobacter jejuni*, *C. coli*, *C. lari*, *C. upsaliensis*, and *C. fetus* subsp. fetus. J. Clin. Microbiol..

[13] Rafailidis P.I., Kofteridis D. (2022). Proposed amendments regarding the definitions of multidrug-resistant and extensively drug-resistant bacteria. Expert. Rev. Anti-Infect. Ther..

[14] Eatemadi A., Risi A.E., Kasliwal A.K., Al-zaabi A.T., Moradzadegan H., Aslani Z. (2021). A Proposed Evidence-Based Local Guideline for Definition of Multidrug-Resistant (MDR), Extensively Drug-Resistant (XDR) and Pan Drug-Resistant (PDR) Bacteria by the Microbiology Laboratory. Int. J. Curr. Sci. Res. Rev..

[15] Xie J., Chen Y., Cai G., Cai R., Hu Z., Wang H. (2023). Tree Visualization by One Table (tvBOT): A web application for visualizing, modifying and annotating phylogenetic trees. Nucleic Acids Res..

[16] Dai L., Sahin O., Grover M., Zhang Q.J. (2020). New and alternative strategies for the prevention, control, and treatment of antibiotic-resistant *Campylobacter*. Transl. Res..

[17] Linn K.Z., Furuta M., Nakayama M., Masuda Y., Honjoh K.I., Miyamoto T. (2021). Characterization and antimicrobial resistance of *Campylobacter jejuni* and *Campylobacter coli* isolated from chicken and pork. Int. J. Food Microbiol..

[18] Asakura H., Sakata J., Nakamura H., Yamamoto S., Murakami S. (2019). Phylogenetic diversity and antimicrobial resistance of *Campylobacter coli* from humans and animals in Japan. Microbes Environ..

[19] Bort B., Martí P., Mormeneo S., Mormeneo M., Iranzo M. (2022). Prevalence and antimicrobial resistance of *Campylobacter* spp. isolated from broilers throughout the supply chain in Valencia, Spain. Foodborne Pathog. Dis..

[20] Dramé O., Leclair D., Parmley E.J., Deckert A., Ouattara B., Daignault D., Ravel A. (2020). Antimicrobial resistance of *Campylobacter* in broiler chicken along the food chain in Canada. Foodborne Pathog. Dis..

[21] Zhang D., Zhang X., Lyu B., Tian Y., Huang Y., Lin C., Yan H., Jia L., Qu M., Wang Q. (2023). Genomic analysis and antimicrobial resistance of *Campylobacter jejuni* isolated from diarrheal patients—Beijing municipality, China, 2019–2021. China CDC Wkly.

[22] Li X., Xu X., Chen X., Li Y., Guo J., Gao J., Jiao X., Tang Y., Huang J. (2023). Prevalence and genetic characterization of *Campylobacter* from clinical poultry cases in China. Microbiol. Spectr..

[23] Espinoza N., Rojas J., Pollett S., Meza R., Patiño L., Leiva M., Camiña M., Bernal M., Reynolds N.D., Maves R. (2020). Validation of the T86I mutation in the *gyrA* gene as a highly reliable real time PCR target to detect Fluoroquinolone-resistant *Campylobacter jejuni*. BMC Infect. Dis..

[24] Aksomaitiene J., Novoslavskij A., Malakauskas M. (2023). Whole-Genome Sequencing-Based Profiling of Antimicrobial Resistance Genes and Core-Genome Multilocus Sequence Typing of *Campylobacter jejuni* from Different Sources in Lithuania. Int. J. Mol. Sci..

[25] Aleksić E., Miljković-Selimović B., Tambur Z., Aleksić N., Biočanin V., Avramov S. (2021). Resistance to Antibiotics in Thermophilic *Campylobacters*. Front. Med..

[26] Mouftah S.F., Cobo-Díaz J.F., Álvarez-Ordóñez A., Elserafy M., Saif N.A., Sadat A., El-Shibiny A., Elhadidy M. (2021). High-throughput sequencing reveals genetic determinants associated with antibiotic resistance in *Campylobacter* spp. from farm-to-fork. PLoS ONE.

[27] Hur J.I., Kim J., Ryu S., Jeon B. (2022). Phylogenetic association and genetic factors in cold stress tolerance in *Campylobacter jejuni*. Microbiol. Spectr..

[28] Yang H., Li Y., Zhang Y., Dong B., Duan B., Guo L., Wang T., Lv X., Zheng M., Cui X. (2023). Prevalence, drug resistance spectrum and virulence gene analysis of *Campylobacter jejuni* in broiler farms in central Shanxi, China. Poult. Sci..

[29] Tang Y., Jiang Q., Tang H., Wang Z., Yin Y., Ren F., Kong L., Jiao X., Huang J. (2020). Characterization and prevalence of *Campylobacter* spp. from broiler chicken rearing period to the slaughtering process in eastern China. Front. Vet. Sci..

[30] Mäesaar M., Meremäe K., Ivanova M., Roasto M. (2018). Antimicrobial resistance and multilocus sequence types of *Campylobacter jejuni* isolated from Baltic broiler chicken meat and Estonian human patients. Poult. Sci..

[31] Reddy S., Zishiri O.T. (2018). Genetic characterisation of virulence genes associated with adherence, invasion and cytotoxicity in *Campylobacter* spp. isolated from commercial chickens and human clinical cases. Onderstepoort J. Vet. Res..

[32] Le L.H.M., Elgamoudi B., Colon N., Cramond A., Poly F., Ying L., Korolik V., Ferrero R.L. (2024). *Campylobacter jejuni* extracellular vesicles harboring cytolethal distending toxin bind host cell glycans and induce cell cycle arrest in host cells. Microbiol. Spectr..

