# Supplementary material for: Antimicrobial Resistance and Genomic Characterization of Campylobacter jejuni and Campylobacter coli Isolated from Retail Chickens in Beijing, China
Source: Microorganisms. 2024 Aug 6;12(8):1601. doi: 10.3390/microorganisms12081601 (PMC11356118; doi:10.3390/microorganisms12081601)
Supplement: Supplementary file 1 [file microorganisms-12-01601-s001.zip › microorganisms-3046037-supplementary.pdf]

microorganisms

MDPI

Article

Antimicrobial Resistance and Genomic Characterization of *Campylobacter jejuni* and *Campylobacter coli* Isolated from Retail Chickens in Beijing, China

Yao Bai [1,†], Jiaqi Ma [2,†], Fengqin Li [1], Baowei Yang [2], Xiu Ren [3], Yeru Wang [1], Yujie Hu [1], Yinping Dong [1], Wei Wang [1], Jing Zhang [1], Shaofei Yan [1,*] and Shenghui Cui [3,*]

[1] NHC Key Laboratory of Food Safety Risk Assessment, China National Centre for Food Safety Risk Assessment, Beijing 100022, China; baiyao@cfsa.net.cn (Y.B.)
[2] College of Food Science and Engineering, Northwest Agriculture and Forestry Science and Technology University, Shaanxi 712100, China; mjq1531900880@nwafu.edu.cn (J.M.)
[3] National Institutes for Food and Drug Control, Beijing 100050, China
[*] Correspondence: yanshaofei@cfsa.net.cn (S.Y.); cuishenghui@aliyun.com (S.C.); Tel.: +86-10-52165596 (S.Y.); +86-10-67095601 (S.C.)
[†] The two authors contributed equally to this study.

Supplementary Materials

Table S1. Relationship between Susceptibility/resistance Patterns and Associated Antibiotic Resistance Genes and point mutations in *C. coli* isolates.

	GEN		ERY		CHL		DC		TET	
	OR	*p*-value	OR	*p*-value	OR	*p*-value	OR	*p*-value	OR	*p*-value
aac(6′)-aph(2′′)	1.0118E+26	0.999	4568241710	1	3.299	1	2.1625E+27	0.999	1.2584E+17	0.999
aadE-Cc	3795044.065	1	1989693.387	1	0	0.999	0.914	1	323094968.6	0.999
ant(6)-Ia	54861221.88	1	2.3834E+15	0.999	75297861.22	1	114224890	1	0.754	1
aph(2′′)-If	0	0.998	0.635	0.784	0	0.999	0	0.999	0	0.999
aph(3′)-III	0	0.999	0	0.998	0	0.998	0	0.998	0.371	1
erm(B)	0	0.999	70646893.47	0.998	0	0.999	0	0.999	0	0.999
cat	871838322.8	0.999	3.821	0.423	1.2931E+17	0.998	0	0.999	0	0.999
fexA	0	1	0	1	1014835424	1	0.243	1	560937444.5	1
tet(L)	0	1	0	1	1014835424	1	0.243	1	560937444.5	1
tet(O)	0	0.999	0	0.999	10.727[a]	0.024	1.871	0.502		
bla$_{OXA-193}$	0	0.999	0	0.998	0	1	0	0.998	0	0.999
bla$_{OXA-489}$	0	0.999	0	0.999	27760879868	1	0	0.999	0	0.999
RpsL (K43R)	0	1	180416521.8	1	3.598	1	2.157	1	2.074	1
23S (A2075G)	0	0.999	0	0.999	0	0.999	0	0.999	0	1
L4 V121A	632801535.5	0.999	272171667.3	0.999	0.428	1	2.666	1	1.44	1
L4 M192I	0	0.999	0	0.999	27760879868	1	0	0.999	0	0.999

GEN: gentamicin, ERY: erythromycin, CHL: chloramphenicol, DC: doxycycline, TET: tetracycline, OR: Odds ratio [a] Indicates an association between antibiotics resistance and the associated-antibiotic resistance gene. [b] Indicates an association between antimicrobial susceptibility and the associated-antibiotic resistance gene. Antimicrobial resistance is defined as an OR > 1 and $p < 0.05$, and antimicrobial susceptibility is defined as an OR < 1 and $p < 0.05$.

Table S2. Relationship between Susceptibility/resistance Patterns and Associated Antibiotic Resistance Genes and point mutations in *C. jejuni* isolates.

	GEN		ERY		CHL		CIP		DC		TET	
	OR	*p*-value	OR	*p*-value	OR	*p*-value	OR	*p*-value	OR	*p*-value	OR	*p*-value
ant(6)-Ia	1.792	1	4.386	1	1.968	1	0	0.999	0.888	1	0.001	1
aph(2'')-If	1.0137E+16	0.998	52.495[a]	0.023	1.7113E+16	0.998	0.276	1	0	0.999	7.609	1
aph(3')-III	16.812	1	16731979.27	0.999	16.376	1	247665265.071	1	4.313	1	0	1
cat	16.812	1	16731979.27	0.999	16.376	1	247665265.071	1	4.313	1	0	1
tet(O)	0.46	0.455	20.541[a]	0.026	0.503	0.445	0	0.999	0.705	0.653	1.5268E+15	0.997
bla$_{OXA-184}$	11.428	1	133379917.5	1	11.395	1	5.2048E+17	0.999	4.995	1	0.173	1
bla$_{OXA-185}$	0.288	1	0	1	0.516	1	31610E+10	1	0	0.999	0	1
bla$_{OXA-193}$	207840075.4	1	1070569937	1	365844883.9	1	2.100E+10	1	1277728214	1	1134099.446	1
bla$_{OXA-465}$	1.121	1	0.459	1	2.02	1	0	1	0	0.999	0	1
RpsL (K43R)	0	1	0	0.999	0	1	1.856	1	6.342	0.301	0	0.999
L4 V82I	0	0.999	0	0.999	0	0.999	0	0.999	2042499100	0.999	0	0.999
L4 T91K	0	1	0	1	0	1	0	1	0	0.998	0	0.999
L4 V121A	2.591	0.5	0.453	0.537	1.472	0.772	242392669.437	0.999	988041491.1	0.999	11368174.31	1
L4 V176I	0	1	0	1	0	1	0	1	0	0.998	0	0.999
L4 T177S	0	1	0	1	0	1	0	1	0	0.998	0	0.999
L4 M192I	10.333[a]	0.001	2.019	0.253	8.286[a]	0.03	2.585	0.697	0.636	0.458	2.270	0.466
GyrA (S22G)	0.19	0.282	0.113	0.247	0.319	0.45	0	0.999	0	0.998	0	0.999
GyrA (T86I)	1336944567	0.999	979075588.4	0.999	1555642433	0.999	1236642433	1	0.207	0.171	4.0700E-09	0.999

GEN: gentamicin, ERY: erythromycin, CHL: chloramphenicol, CIP: ciprofloxacin, DC: doxycycline, TET: tetracycline, OR: Odds ratio [a] Indicates an association between antibiotics resistance and the associated-antibiotic resistance gene. [b] Indicates an association between antimicrobial susceptibility and the associated-antibiotic resistance gene. Antimicrobial resistance is defined as an OR > 1 and $p < 0.05$, and antimicrobial susceptibility is defined as an OR < 1 and $p < 0.05$.

Table S3. Relationship between Susceptibility/resistance Patterns and Associated Virulence Genes in *C. coli* isolates.

	GEN		ERY		CHL		DC		TET	
	OR	*p*-value	OR	*p*-value	OR	*p*-value	OR	*p*-value	OR	*p*-value
cheA	756362537.1	1	1150324865	1	323618950.7	1	1263792058	1	0	1
Cj1135	1.017	0.982	0.251	0.166	0.897	0.88	0.037[b]	0.001	0.547	0.514
htrB	2.341	0.602	0.053	0.094	1.986	0.643	9.017	0.153	180178356.7	0.999
kpsM	0.09[b]	0.002	0.098[b]	0.003	0.167[b]	0.035	0.307	0.102	2.279	0.472
flaA	0	1	0	1	0	1	0	1	0	1
flaB	311040269.3	1	221852055.5	1	1343515266	1	633182942.5	1	322034793.9	1

GEN: gentamicin, ERY: erythromycin, CHL: chloramphenicol, DC: doxycycline, TET: tetracycline, OR: Odds ratio [a] Indicates an association between antibiotics resistance and the associated-virulence

gene. [b] Indicates an association between antimicrobial susceptibility and the associated-virulence gene. Antimicrobial resistance is defined as an OR > 1 and $p < 0.05$, and antimicrobial susceptibility is defined as an OR < 1 and $p < 0.05$.

Table S4. Relationship between Susceptibility/resistance Patterns and Associated Virulence genes in *C. jejuni* isolates.

	GEN		ERY		CHL		CIP		DC		TET	
	**OR**	***p*-value**	**OR**	***p*-value**	**OR**	***p*-value**	**OR**	***p*-value**	**OR**	***p*-value**	**OR**	***p*-value**
porA	0.41	0.208	0.296	0.142	0.412	0.196	3.942	0.216	0.832	0.777	0.227	0.226
Cj1135	5.139[a]	0.036	23.801[a]	0.009	2.659	0.156	13.316	0.38[a]	1.345	0.636	10.214[a]	0.017
kpsM	0.942	1	2587778067	1	0.703	1	0.532	1	1620050814	1	1.91	1
NeuA1	0.843	0.909	0.818	0.905	0.814	0.889	93267465.85	0.999	1531394669	0.999	87609726.93	0.999
wlaN	0.843	0.909	0.818	0.905	0.814	0.889	93267465.85	0.999	1531394669	0.999	87609726.93	0.999
flaA	1.129	0.838	0.39	0.154	2.214	0.171	6.430	0.136	0.994	0.992	0.338	0.263
flaB	1.129	0.838	0.39	0.154	2.214	0.171	6.430	0.136	0.994	0.992	0.338	0.263
cdtA	0	0.999	0.665	0.787	0	0.999	0	0.999	0.965	0.98	0	0.999
cdtB	2.4579E+18	0.999	2587781473	1	1.8347E+18	0.999	0.532	1	1620050956	1	1.91	1
cdtC	2.4579E+18	0.999	2587781473	1	1.8347E+18	0.999	57886525.140	1	1620050956	1	1.91	1

GEN: gentamicin, ERY: erythromycin, CHL: chloramphenicol, DC: doxycycline, TET: tetracycline, OR: Odds ratio [a] Indicates an association between antibiotics resistance and the associated-virulence gene. [b] Indicates an association between antimicrobial susceptibility and the associated-virulence gene. Antimicrobial resistance is defined as an OR > 1 and $p < 0.05$, and antimicrobial susceptibility is defined as an OR < 1 and $p < 0.05$.

Table S5. Reference ARGs sequences used in this study.

ID	ARG	Sequence (5′-3′)
M13771	*aac(6′)-aph(2′′)_1*	ATGAATATAGTTGAAAATGAAATATGTATAAGAACTTTAATAGATGATGATTTTCCTTTGATGTTAAAATGGTTAACT<br>GATGAAAGAGTATTAGAATTTTATGGTGGTAGAGATAAAAAATATACATTAGAATCATTAAAAAAACATTATACAG<br>AGCCTTGGGAAGATGAAGTTTTTAGAGTAATTATTGAATATAACAATGTTCCTATTGGATATGGACAAATATATAAA<br>ATGTATGATGAGTTATATACTGATTATCATTATCCAAAAACTGATGAGATAGTCTATGGTATGGATCAATTTATAGG<br>AGAGCCAAATTATTGGAGTAAAGGAATTGGTACAAGATATATTAAATTGATTTTTGAATTTTTGAAAAAAGAAAGA<br>AATGCTAATGCAGTTATTTTAGACCCTCATAAAAATAATCCAAGAGCAATAAGGGCATACCAAAAATCTGGTTTTA<br>GAATTATTGAAGATTTGCCAGAACATGAATTACACGAGGGCAAAAAAGAAGATTGTTATTTAATGGAATATAGATA<br>TGATGATAATGCCACAAATGTTAAGGCAATGAAATATTTAATTGAGCATTACTTTGATAATTTCAAAGTAGATAGTA<br>TTGAAATAATCGGTAGTGGTTATGATAGTGTGGCATATTTAGTTAATAATGAATACATTTTTAAAACAAAATTTAGTA<br>CTAATAAGAAAAAAGGTTATGCAAAAGAAAAAGCAATATATAATTTTTTAAATACAAATTTAGAAACTAATGTAAA<br>AATTCCTAATATTGAATATTCGTATATTAGTGATGAATTATCTATACTAGGTTATAAAGAAATTAAAGGAACTTTTTT<br>AACACCAGAAATTTATTCTACTATGTCAGAAGAAGAACAAAATTTGTTAAAACGAGATATTGCCAGTTTTTTAAGAC<br>AAATGCACGGTTTAGATTATACAGATATTAGTGAATGTACTATTGATAATAAACAAAATGTATTAGAAGAGTATATA<br>TTGTTGCGTGAAACTATTTATAATGATTTAACTGATATAGAAAAAGATTATATAGAAAGTTTTATGGAAAGACTAAA<br>TGCAACAACAGTTTTTGAGGGTAAAAAGTGTTTATGCCATAATGATTTTAGTTGTAATCATCTATTGTTAGATGGCAA<br>TAATAGATTAACTGGAATAATTGATTTTGGAGATTCTGGAATTATAGATGAATATTGTGATTTTATATACTTACTTGA<br>AGATAGTGAAGAAGAAATAGGAACAAATTTTGGAGAAGATATATTAAGAATGTATGGAAATATAGATATTGAGAA<br>AGCAAAAGAATATCAAGATATAGTTGAAGAATATTATCCTATTGAAACTATTGTTTATGGAATTAAAAATATTAAAC<br>AGGAATTTATCGAAAATGGTAGAAAAGAAATTTATAAAAGGACTTATAAAGATTGA
CP013733	*aadE-Cc_1*	ATGCAAAATCAAGATAAATTTTTAAAACAATTTAAAAAATTAGCACTTTTAGATAAAAATATACGCCTTGTTACACT<br>TGAAGGCTCAAGGGTTAATAAAAAAGCGAAGAAAGATAAATATCAAGACTATGATATTTCATTTTTTGTGCCACTTG<br>ATAAAATGAAAGATTTTTTAGGGCTTAATGAAAAGCAAAATTTTAACGAATGTAAAAACTTGCCAAAATGTATTTTA<br>GAGCTTGAAAAATCTTCATATTTTAAAAAAATTTTAATGCTTCAAATGCCTGAATGTATGGAGTTTTATCCACCTGAT<br>TTGCCACAAAATTGGATAAGTTTTTTAGTGCTTTTTGAAAGTGGAGTGAGGCTTGATTTAACCATTATTCCTTTAGAA<br>GATTTGAAAAATTACTATGAATTTGAGCCTTTAAGCCAAGCGCTTTTGGATAAAAATGGGCTTTTTACGCACACTATT<br>CCAAAAGCCCCATTTAGCATCACACACCTTAGCCAAAGAAGCTTTGATGATGTTTGCAATGAGTTTTATTTTCTTTAT<br>AGTTGCTTAAAAAAAGCTCTTTTAAGAAAGCAGTTTATTTTGTCAAATCATTTGCTAAATTCTTTGAGAAAAGCACTT<br>TTTGATTTGCTTAGTTTTAAAATTGGCTTAAATTTTGGCTTTGAAATCTGGCTGGGAAAAGAATACACTAATATTTTA<br>GAATTTTTAGAAGAAAAAGAAGTAAAAATCATCTTAAAATCTTTTAACACCGCCACGCTAGAACACATCAAAAAA<br>GCAAGAAAAAAGCTTGAAATTTTATTTCATAAAAATGCTAAATTTGTAGCAAAAAAGAGTGATTTTAAGCTTTTTCC<br>TTACCGAAAAAAATGTGAAAAGGTATTGTAAAATTTTAGGAAAATTGTAA

AF330699	*ant(6)-Ia_1*	ATGAGATCAGAAAAAGAAATGATGGATTTAGTACTTTCTTTAGCAGAACAGGATGAACGTATTCGAATTGTGACCC<br>TTGAGGGGTCACGCGCAAATATTAATATACCTAAAGATGAATTTCAGGATTATGATATTACATATTTTGTAAGTGAT<br>ATAGAACCGTTTATATCTAATGATGACTGGCTTAATCAATTTGGGAATATAATAATGATGCAAAAGCCGGAGGATAT<br>GGAATTATTCCCACCTGAAGAAAAGGGATTTTCCTATCTTATGCTATTTGATGATTACAATAAAATTGATCTTACCTT<br>ATTGCCCTTGGAAGAGTTAGATAATTACCTAAAGGGCGATAAATTAATAAAGGTTCTAATTGATAAAGATTGTAGA<br>ATTAAAAGGGACATAGTTCCGACTGATATAGATTATCATGTAAGAAAGCCAAGCGCAAGGGAGTATGATGATTGCT<br>GCAATGAATTTTGGAATGTAACACCTTATGTTATTAAAGGATTGTGCCGTAAGGAAATTTTATTTGCTATTGATCATT<br>TTAATCAGATTGTTCGCCATGAGCTGCTGAGAATGATATCATGGAAGGTCGGCATCGAAACAGGCTTTAAATTAAGT<br>GTAGGCAAGAACTATAAGTTTATTGAAAGGTATATATCCGAGGATTTGTGGGAGAAACTTTTGTCCACCTACCGGAT<br>GGATTCCTATGAAAACATATGGGAAGCATTATTTCTATGCCATCAATTGTTCAGGGCGGTATCCGGTGAGGTGGCGG<br>AAAGGCTTCATTATGCCTATCCGGAGTATGATAGGAATATAACAAAATATACCAGGGACATGTATAAAAAATACAC<br>TGGTAAAACCGGCTGCCTGGATAGCACATATGCCGCTGATATAGAAGAGAGGCGGGAACAGTGA
KF864551	*ant(6)-Ia_3*	ATGAGATCAGAAAAGGAAGTTTATGATATTGTTTTGAATTTTGCAAAAACAGACAAACGCATTCGCATGGTTACTTT<br>GGAAGGATCTAGAACAAATACAAATATTCCGCCTGATGATTTTCAGGATTTTGATATTACTTTTTTTGTTACGGATAT<br>GGACAGCTTCACAAGTGATGATAAATGGCTAGATATATTTGGTGAAAGGTTGATTCTGCAAAAGCCGGAAGATATG<br>GAATTATTTCCAGCTGTAGAAAAGGGATTTTCATATTTAATGCTGTTTACTGATGATGTTAAGATAGATTTAACTTTG<br>CTGCCGCTGGAACTGATAGACGAGTATTTTACATGGGATAAACTGGTAAAGTTACTGTTGGATAAAGACAACCGTA<br>TCGTAAAGCCGCCAATACCAACGGATATAGACTACCACTTGCAGAAGCCTACTCAAAGAATGTTTGACGATTGCTG<br>TAATGAATTTTGGAATACTACAACATATGTAGTAAAGGGCTTATGCCGCAAAGAAATTCTTTTTGCTATTGACCATAT<br>GAATGATATAGTACGAAAAGAATTGCTTCGCATGATTTCCTGGCTGATTGGTATCAAACAGGGATTTCATTTCAGTTT<br>GGGAAAAAACTATAAATTTATGAAGCAATATGTCCCAGAGGAATTGTGGGAACGACTTATGTCCACTTATAATATG<br>GATTCCTATCCCCATATGTGGGAATCCTTTGAACAATGTATGGCATTGTTCCGGGAGGTTTCGTCAGAAGTGGCATG<br>CCAGTTGGATTACCAGTATCCACTATATGATGAAAAAATCAGTAATTATGTGATTCGGCAAAAGAAAAAATATGGC<br>ATTGAAGATGATAACAAATAA
AY701528	*aph(2'')-If_2*	ATGGATATAAAAAAGATAATAGAAGAAAAATGCAATATAGTTGTTGATAGTATAAAGTTGATTGGTGAGGGTTATG<br>ACAGCAAAGCATACATTGTAAATAATGAATATGTTTTCAAAATCAAATTTAGTGCTAATAAGAAAAAAGGGTATGA<br>AAAAGAAAAAGCAATATATGATTTTCTAAACAAAAAATTAAATACAAATATTAAAATACCAAATATAGAATATTCA<br>TATATAAGTGAAGAATTATCTATTTTAGGATATAAAGAAATTAAAGGAACTTTTTTAACACCAGAAATATATTTTGC<br>CTTATCAAAAGAAAAGCAAGAATTATTAAAGCAAGATATTGCTATGTTTTTAAGACAAATGCACGATTTAGATTATA<br>GTGAAATAAGTTCATATACGATAGACAATAAACAAAATGTTTTAGAAGAATATCAATTACTTAAAGAAACAATATA<br>TGATAGTCTTACTGATATTGAAAAACAATATGTAGAAGAATTTATGCAAAGATTAAATAGTACAACTATATTTGATG<br>GTAAAAAATGCTTATGCCATAATGATTTTAGTTGTAATCATTTATTACTTGATGATGAAAATAGATTATGTGGTGTAA<br>TAGATTTTGGAGATTCTGGAATTATAGATGAATACTGTGATTTCATATATTTGCTAGAAGATAGTGAAGAAGAAATT

		GGCGTGTCTTTTGGAGAAGATATATTAAGATTATACGGAAATATTGATATTTCAAAAGCAAAGGAATATCAAGATGT<br>TGTAGAACAATATTATCCAATAGAAACTATTGTATATGGTATTAAAAATAATAGACCTGATTTTATAGAAAAAGGTA<br>GAAAAGAGATTTATATAAGAACTCGCAAAGATGAAAAATTAAGGAAGTGA
M26832	*aph(3′)-III_1*	ATGGCTAAAATGAGAATATCACCGGAATTGAAAAAACTGATCGAAAAATACCGCTGCGTAAAAGATACGGAAGGA<br>ATGTCTCCTGCTAAGGTATATAAGCTGGTGGGAGAAAATGAAAACCTATATTTAAAAATGACGGACAGCCGGTATA<br>AAGGGACCACCTATGATGTGGAACGGGAAAAGGACATGATGCTATGGCTGGAAGGAAAGCTGCCTGTTCCAAAGG<br>TCCTGCACTTTGAACGGCATGATGGCTGGAGCAATCTGCTCATGAGTGAGGCCGATGGCGTCCTTTGCTCGGAAGAG<br>TATGAAGATGAACAAAGCCCTGAAAAGATTATCGAGCTGTATGCGGAGTGCATCAGGCTCTTTCACTCCATCGACA<br>TATCGGATTGTCCCTATACGAATAGCTTAGACAGCCGCTTAGCCGAATTGGATTACTTACTGAATAACGATCTGGCC<br>GATGTGGATTGCGAAAACTGGGAAGAAGACACTCCATTTAAAGATCCGCGCGAGCTGTATGATTTTTTAAAGACGG<br>AAAAGCCCGAAGAGGAACTTGTCTTTTCCCACGGCGACCTGGGAGACAGCAACATCTTTGTGAAAGATGGCAAAGT<br>AAGTGGCTTTATTGATCTTGGGAGAAGCGGCAGGGCGGACAAGTGGTATGACATTGCCTTCTGCGTCCGGTCGATCA<br>GGGAGGATATCGGGGAAGAACAGTATGTCGAGCTATTTTTTGACTTACTGGGGATCAAGCCTGATTGGGAGAAAAT<br>AAAATATTATATTTTACTGGATGAATTGTTTTAG
JQ396378	*bla*$_{\text{OXA-184\_1}}$	TTGAAAAAAATACTTTTACTTTTTAGTCTTTTTTACTCTTTTGCTTTGGCAAATGATAAATTAAAAGATTTTTTTAAAG<br>ACTACAATACAAGCGGAGTTTTTATAACTTTTGATGGAAAACATTATGCAAGTAATAATTTTAAAAGAGCTAAAGA<br>ACCTTTTTCTCCTGCTTCGACTTTTAAAATTTTTAATGCTTTAATTGCGCTTGATAATGGTGTAGTTAAAGATACAAAG<br>GAAATTTTTTATCATTATAAGGGTGAAAAAGTATTTTTGCCCTCTTGGAAACAAGATGCTAGTTTAAGCTCAGCCATA<br>AAACGCTCTCAAGTGCCTGCTTTTAAAGAATTGGCAAGAAAAATAGGACTTAAAACCATGCAAGAAAGCTTAAATA<br>AACTTTCCTATGGAAATACAAAAATTTCAAAAATCGATACCTTTTGGTTGGATAATTCTTTACAAATTTCTGCAAAAA<br>ATCAAGCTGATTTGCTTTTTAAACTTTCACAAAATTCTTTACCTTTTTCCAAGAAAAGTCAAGAAGAAGTTAAAAAA<br>ATTATTCTTTTTAAAGAAGATAAAATCCAAAAAATTTATGCTAAAACAGGTTTTAATGATGGTATAAATTTGGCTTG<br>GATTGTTGGATTTATAGAGAGTAAAAACAAAATTTTATCTTTTGCCTTAAATGTTGATATAAAGAACATTAAAAATC<br>TTAAAATAAGAGAAGAATTGCTAGAAAAATATATTTATTCTTTAAACTAA
JQ396379	*bla*$_{\text{OXA-185\_1}}$	TTGAAAAAAATACTTTTACTTTTTAGTCTTTTTTACTCTTTTGCTTTGGCAAATGATAAATTAAAAGATTTTTTTAAAG<br>ACTACAATACAAGCGGAGTTTTTATAACTTTTGATGGAAAACATTATGCAAGTAATAATTTTAAAAGAGCTAAAGA<br>ACCTTTTTCTCCTGCTTCGACTTTTAAAATTTTTAATGCTTTAATTGCGCTTGATAATGGTGTAGTTAAAGATACAAAG<br>GAAATTTTTTATCATTATAAGGGTGAAAAAGTATTTTTGCCCTCTTGGAAACAAGATGCTAGTTTAAGCTCAGCCATA<br>AAACGCTCTCAAGTGCCTGCTTTTAAAGAATTGGCAAGAAAAATAGGACTTAAAACCATGCAAGAAAGCTTAAATA<br>AACTTTCCTATGGAAATACAAAAATTTCAAAAATCGATACCTTTTGGTTGGATAATTCTTTACAAATTTCTGCAAAAA<br>ATCAAGCTGATTTGCTTTTTAAACTTTCACAAAATTCTTTACCTTTTTCCAAGAAAAGTCAAGAAGAAGTTAAAAAA<br>ATTATTCTTTTTAAAGAAGATAAAATCCAAAAAATTTATGCTAAAACAGGTTTTAATGATGGTATAAATTTGGCTTG

		GATTGTTGGATTTATAGAGAGTAAAAACAAAATTTTATCTTTTGCCTTAAATGTTGATATAAAGAACATTAAAAATA<br>TTAAAATAAGAGAAGAATTGCTAGAAAAATATATTTATTCTTTAAACTAA
CP013032	*bla*$_{OXA-193\_1}$	ATGAAAAAAATAACTTTATTTTTGCTTTTCTTAAATTTAGTGTTTGGGCAAGATAAGATATTAAATAATTGGTTTAAA<br>GAGTATAATACAAGCGGCACTTTTGTTTTTTATGATGGAAAAACTTGGGCGAGTAACGACTTTTCAAGGGCTATGGA<br>GACTTTCTCTCCCGCTTCCACTTTTAAAATTTTTAATGCTCTAATTGCACTTGATAGTGGTGTGATAAAAACTAAAAA<br>AGAAATTTTTTATCACTATAGAGGTGAAAAAGTATTTTTATCTTCTTGGGCGCAAGATATGAATTTAAGTTCAGCTAT<br>AAAATATTCTAATGTTCTTGCTTTTAAAGAAGTGGCAAGAAGAATTGGTATCAAAACTATGCAAGAATATTTAAACA<br>AGCTTCATTATGGTAATGCTAAAATTTCCAAGATCGATACTTTTTGGCTTGACAACTCACTAAAAATAAGCGCTAAA<br>GAACAAGCAATTTTGCTTTTTAGACTTTCACAAAATAGCTTACCTTTTTCTCAAGAAGCAATGAATAGTGTTAAGGA<br>AATGATTTATTTAAAAAATATGGAAAATTTAGAGCTTTTTGGAAAAACAGGTTTTAATGATGAGCAAAAAATTGCTT<br>GGATTGTAGGTTTTGTGTATTTAAAAGATGAAAATAAATATAAGGCTTTCGCGCTAAATTTAGATATTGATAAATTTG<br>AAGATTTATATAAAAGAGAAAAAATTTTAGAAAAATATTTAGATGAACTTGTAAAAAAAGTTAAAAATGATGGCTA<br>G
KR061500	*bla*$_{OXA-465\_1}$	TTGAAAAAAATACTTTTACTTTTTAGTCTTTTTTACTCTTTTGCTTTGGCAAATGATAAATTAAAAGATTTTTTTAAAG<br>ACTACAATACAAGCGGAGTTTTTATAACTTTTGATGGAAAACATTATGCAAGTAATGATTTTAAAAGAGCTAAAGA<br>ACCTTTTTCTCCTGCTTCGACTTTTAAAATTTTTAATGCTTTAATTGCGCTTGATAACGGTGTAGTTAAAGATACAAAG<br>GAAATTTTTTATCATTATAAGGGTGAAAAAGTATTTTTGCCTTCTTGGAAACAAGATGCTAGTTTAAGCTCAGCCATA<br>AAACGCTCTCAAGTGCCTGCTTTTAAAGAATTGGCAAGAAAAATAGGACTTAAAACCATGCAAGAAAGCTTAAATA<br>AACTTTCCTATGGAAATGCAAAAATTTCAAAAATCGATACCTTTTGGTTGGATAATTCTTTACAAATTTCTGCAAAAA<br>ATCAAGCTGATTTGCTTTTTAAACTTTCACAAAATTCTTTACCTTTTTCCAAGAAAAGTCAAGAAGAAGTTAAAAAA<br>ATTATTCTTTTTAAAGAAGATAAAATCCAAAAAAATTTATGCTAAAACAGGTTTTAATGATGGTATAAATTTGGCTTG<br>GATTGTCGGATTTATAGAGAGTAAAAACAAAATTTTATCTTTTGCCTTAAATGTTGATATAAAGGACATTAAAAATA<br>TTAAAATAAGAGAAGAATTGCTAGAAAAATATATTTATTCTTTAAACTAA
CP013733	*bla*$_{OXA-489\_1}$	ATGAAAAAAATAACTTTATTTTTACTTTTCTTAAATTTAGTGTTTGGGCAAGATAAGATATTAAATAATTGGTTTAAA<br>GAGTATAATACAAGCGGCACTTTTGTTTTTTATGATGGAAAAACTTGGGCGAGTAACGACTTTTCAAGGGCTATGGA<br>GACTTTCTCTCCCGCTTCCACTTTTAAAATTTTTAATGCTCTAATTGCACTTGATAGTGGTGTGATAAAAACTAAAAA<br>AGAAATTTTTTATCACTATAGAGGTGAAAAAGTATTTTTATCTTCTTGGGCGCAAGATATGAATTTAAGTTCAGCTAT<br>AAAATATTCTAATGTTCTTGCTTTTAAAGAAGTGGCAAGAAGAATTGGTATCAAAACTATGCAAGAATATTTAGACA<br>AGCTTCATTATGGTAATGCTAAAATTTCCAAGATCGATACTTTTTGGCTTGACAACTCACTAAAAATAAGCGCTAAA<br>GAACAAGCAATTTTGCTTTTTAGACTTTCACAAAATAGCTTACCTTTTTCTCAAGAAGCAATGAATAGTGTTAAGGA<br>AATGATTTATTTAAAAAATATGGAAAATTTAGAGCTTTTTGGAAAAACAGGTTTTAATGATGAGCAAAAAATTGCTT<br>GGATTGTAGGTTTTGTGTATTTAAAAGATGAAAATAAATATAAGGCTTTCGCGCTAAATTTAGATATTGATAAATTTG

		AAGATTTATATAAAAGAGAAAAAATTTTAGAAAAATATTTAGATGAACTTGTAAAAAAAGTTAAAAATGATGGCTAG
M35190	*cat_2*	ATGCAATTCACAAAGATTGATATAAATAATTGGACACGAAAAGAGTATTTCGACCACTATTTTGGCAATACGCCCTGCACATATAGTATGACGGTAAAACTCGATATTTCTAAGTTGAAAAAGGATGGAAAAAAGTTATACCCAACTCTTTTATATGGAGTTACAACGATCATCAATCGACATGAAGAGTTCAGGACCGCATTAGATGAAAACGGACAGGTAGGCGTTTTTTCAGAAATGCTGCCTTGCTACACAGTTTTTCATAAGGAAACTGAAACCTTTTCGAGTATTTGGACTGAGTTTACAGCAGACTATACTGAGTTTCTTCAGAACTATCAAAAGGATATAGACGCTTTTGGTGAACGAATGGGAATGTCCGCAAAGCCTAATCCTCCGGAAAACACTTTCCCTGTTTCTATGATACCGTGGACAAGCTTTGAAGGCTTTAACTTAAATCTAAAAAAAGGATATGACTATCTACTGCCGATATTTACGTTTGGGAAGTATTATGAGGAGGGCGGAAAATACTATATTCCCTTATCGATTCAAGTGCATCATGCCGTTTGTGACGGCTTTCATGTTTGCCGTTTTTTGGATGAATTACAAGACTTGCTGAATAAATAA
X66468	*erm*(B)_18	ATGTATCACTTCAGGAGTGATTACATGAACAAAAATATAAAATATTCTCAAAACTTTTTAACGAATGAAAAGGTACTCAACCAAATAATAAAACAATTGAATTTAAAAGAAACCGATACCGTTTACGAAATTGGAACAGGTAAAGGGCATTTAACGACGAAACTGGCTAAAATAAGTAAACAGGTAACGTCTATTGAATTAGACAGTCATCTATTCAACTTATCGTCAGAAAAATTAAAACTGAACATTCGTGTCACTTTAATTCACCAAGATATTCTACAGTTTCAATTCCCTAACAAACAGAGGTATAAAATTGTTGGGAATATTCCTTACCATTTAAGCACACAAATTATTAAAAAAGTGGTTTTTGAAAGCCATGCGTCTGACATCTATCTGATTGTTGAAGAAGGATTCTACAAGCGTACCTTGGATATTCACCGAACACTAGGGTTGCTCTTGCACACTCAAGTCTCGATTCAGCAATTGCTTAAGCTGCCAGCGGAATGCTTTCATCCTAAACCAAAAGTAAACAGTGTCTTAATAAAACTTACCCGCCATACCACAGATGTTCCAGATAAATATTGGAAGCTATATACGTACTTTGTTTCAAAATGGGTCAATCGAGAATATCGTCAACTGTTTACTAAAAATCAGTTTCATCAAGCAATGAAACACGCCAAAGTAAACAATTTAAGTACCGTTACTTATGAGCAAGTATTGTCTATTTTTAATAGTTATCTATTATTTAACGGGAGGAAATAA
AJ549214	*fexA_1*	AATGAAAAAGGATAGTAAATCTAAAGAAATGATTCAATCTGAAAAAAGGGGTTCTACTAGGCTTTTAATGATGGTACTCTCCCTATCTGTACTTGTAGGTGCAATTACGGCTGATTTAGTCAATCCCGTACTTCCACTAATAAGCAAAGATTTAGAAGCTTCGAAATCTCAAGTGAGTTGGATAGTTAGTGGTATTGCACTTGTTCTTGCGATTGGAGTTCCGATTTATGGTCGAATCTCAGACTTTTTTGAGTTACGAAAGCTATATATCTTTGCCATTATGATTCTGGCAAGTGGTAGTCTTTTATGTGCAATTGCCCCGAACCTCCCATTGTTGGTTTTGGGAAGAATGGTTCAGGGTGCTGGGATGTCCGCAATTCCAGTTCTATCAATCATTGCAATTTCGAAGGTTTTCCCACAAGGAAAACGTGGGGGAGCTTTGGGAATTATCGCAGGAAGTATTGGTGTTGGAACTGCTGCTGGTCCAATATTTGGTGGAGTAGTTGGTCAATATTTAGGGTGGAATGCCTTGTTTTGGTTCACATTTTTGTTAGCCATTATGATTGTTATTGGTGCCTACTACGCGTTACCGACAATTAAACCGGCAGAATCCGTAGGAAGCAATAAGAACTTTGATTTCATTGGTGGTTTATTCCTCGGCCTCACAGTAGGATTACTCCTTTTTGGCATCACTCAAGGAGAAACTTCTGGTTTTTCTTCGTTCTCATCGTTAACTAGCCTAATTGGTTCTGTTGTAGCTTTGGTGGGATTTATTTGGAGAATTGTTACCGCAGAAAATCCATTTGTACCACCTGTCCTGTTCAATAACAAGGATTATGTCAATACGGTCATAATTGCATTTTTTTCGATGTTTGCTTATTTCGCTGTTCTTGTGTTCGTCCCATTACTAGTCGTTGAGGTGAATGGACTCTCTTCT

		GGACAGGCTGGAATGATATTGTTGCCAGGTGGTGTGGCTGTTGCAATCTTATCTCCCTTCGTTGGCCGTCTTTCTGAT<br>CGATTTGGGGATAAACGTCTGATAATTACTGGGATGACTCTGATGGGGCTGTCTACCTTATTCTTGTCCACCTATGCA<br>TCTGGTGCTTCACCTCTGTTAGTTTCCGTGGGGGTCCTCGGAGTAGGGATTGCTTTTGCATTCACGAATTCTCCCGCA<br>AATAACGCCGCAGTAAGTGCACTCGATGCAGACAAGGTTGGTGTCGGAATGGGGATTTTCCAAGGTGCTTTGTACCT<br>TGGAGCAGGAACTGGAGCAGGTATGATTGGAGCATTATTATCCGCTCGACGTGATGCTACTGAGCCGATAAATCCA<br>TTATATATATTGGACGCTATGTCCTACTCAGATGCGTTCCTTGCAGCTACAGGGGCAATACTCATTGCCTTAATAGCT<br>GGATTAGGTTTAAAAAAGCGTGGGTAA
M29725	*tet*(L)_2	GTGAATACATCCTATTCACAATCGAATTTACGACACAACCAAATTTTAATTTGGCTTTGCATTTTATCTTTTTTTAGCG<br>TATTAAATGAAATGGTTTTGAACGTCTCATTACCTGATATTGCAAATGATTTTAATAAACCACCTGCGAGTACAAAC<br>TGGGTGAACACAGCCTTTATGTTAACCTTTTCCATTGGAACAGCTGTATATGGAAAGCTATCTGATCAATTAGGCATC<br>AAAAGGTTACTCCTATTTGGAATTATAATAAATTGTTTCGGGTCGGTAATTGGGTTTGTTGGCCATTCTTTCTTTTCCT<br>TACTTATTATGGCTCGTTTTATTCAAGGGGCTGGTGCAGCTGCATTTCCAGCACTCGTAATGGTTGTAGTTGCGCGCT<br>ATATTCCAAAGGAAAATAGGGGTAAAGCATTTGGTCTTATTGGATCGATAGTAGCCATGGGAGAAGGAGTCGGTCC<br>AGCGATTGGTGGAATGATAGCCCATTATATTCATTGGTCCTATCTTCTACTCATTCCTATGATAACAATTATCACTGTT<br>CCGTTTCTTATGAAATTATTAAAGAAAGAAGTAAGGATAAAAGGTCATTTTGATATCAAAGGAATTATACTAATGTC<br>TGTAGGCATTGTATTTTTTATGTTGTTTACAACATCATATAGCATTTCTTTTCTTATCGTTAGCGTGCTGTCATTCCTGA<br>TATTTGTAAAACATATCAGGAAAGTAACAGATCCTTTTGTTGATCCCGGATTAGGGAAAAATATACCTTTTATGATT<br>GGAGTTCTTTGTGGGGGAATTATATTTGGAACAGTAGCAGGGTTTGTCTCTATGGTTCCTTATATGATGAAAGATGTT<br>CACCAGCTAAGTACTGCCGAAATCGGAAGTGTAATTATTTTCCCTGGAACAATGAGTGTCATTATTTTCGGCTACATT<br>GGTGGGATACTTGTTGATAGAAGAGGTCCTTTATACGTGTTAAACATCGGAGTTACATTTCTTTCTGTTAGCTTTTTAA<br>CTGCTTCCTTTCTTTTAGAAACAACATCATGGTTCATGACAATTATAATCGTATTTGTTTTAGGTGGGCTTTCGTTCAC<br>CAAAACAGTTATATCAACAATTGTTTCAAGTAGCTTGAAACAGCAGGAAGCTGGTGCTGGAATGAGTTTGCTTAACT<br>TTACCAGCTTTTTATCAGAGGGAACAGGTATTGCAATTGTAGGTGGTTTATTATCCATACCCTTACTTGATCAAAGGT<br>TGTTACCTATGGAAGTTGATCAGTCAACTTATCTGTATAGTAATTTGTTATTACTTTTTTCAGGAATCATTGTCATTAG<br>TTGGCTGGTTACCTTGAATGTATATAAACATTCTCAAAGGGATTTCTAA
M18896	*tet*(O)_1	ATGAAAATAATTAACTTAGGCATTCTGGCTCACGTTGACGCAGGAAAGACAACATTAACGGAAAGTTTATTGTATA<br>CCAGTGGTGCAATTGCAGAACTAGGGAGCGTAGATGAAGGCACAACAAGGACAGATACAATGAATTTGGAGCGTC<br>AAAGGGGAATCACTATCCAGACAGCAGTGACATCTTTTCAGTGGGAGGATGTAAAAGTCAACATTATAGATACGCC<br>AGGCCATATGGATTTTTTGGCGGAAGTATACCGTTCTTTATCCGTATTAGACGGAGCAGTATTATTAGTTTCTGCAAA<br>GGATGGCATACAGGCACAGACCCGTATACTGTTTCATGCACTACAGATAATGAAGATTCCGACAATTTTTTTCATCA<br>ATAAAATTGACCAAGAGGGGATTGATTTGCCAATGGTATATCGGGAAATGAAAGCAAAGCTTTCTTCGGAAATTAT<br>AGTGAAGCAAAAGGTTGGGCAGCATCCCCATATAAATGTAACGGACAATGACGATATGGAACAGTGGGATGCGGT<br>AATTATGGGAAACGATGAACTATTAGAGAAATATATGTCAGGGAAACCGTTTAAAATGTCAGAACTGGAACAGGA

		AGAAAACAGGAGATTCCAAAACGGAACGTTATTTCCCGTTTATCACGGAAGCGCTAAAAACAATCTGGGGACTCGG<br>CAGCTTATAGAAGTAATTGCCAGTAAATTTTATTCATCAACGCCTGAAGGTCAATCTGAACTATGCGGGCAGGTTTT<br>TAAGATTGAATATTCAGAGAAAAGGCGGCGTTTTGTTTATGTGCGTATATATAGCGGAACATTGCATTTGAGGGATG<br>TTATTAGAATATCTGAAAAAGAGAAAATAAAAATCACAGAGATGTATGTTCCGACAAACGGTGAATTATATTCATC<br>CGATACAGCCTGCTCTGGTGATATTGTAATTTTACCAAATGATGTTTTGCAGCTAAACAGTATTTTGGGGAACGAAA<br>TACTGTTGCCGCAGAGAAAATTTATTGAAAATCCTCTCCCTATGATCCAAACAACGATTGCAGTAAAGAAATCTGA<br>ACAGCGGGAAATATTGCTTGGGGCACTTACAGAAATTTCAGATTGCGACCCTCTTTTAAAATATTATGTGGATACTA<br>CAACGCATGAGATTATACTTTCTTTTTTGGGGAATGTGCAGATGGAAGTCATTTGTGCCATCCTTGAGGAAAAATAT<br>CATGTGGAGGCAGAAATAAAAGAGCCTACTGTTATATATATGGAAAGACCGCTTAGAAAAGCAGAATATACCATC<br>CACATAGAAGTCCCGCCAAATCCTTTCTGGGCTTCTGTCGGGTTGTCCATAGAGCCGCTCCCTATTGGAAGCGGAGT<br>GCAGTATGAAAGCAGAGTTTCACTTGGATATTTAAATCAATCGTTCCAAAATGCGGTTATGGAGGGGGTTCTTTATG<br>GCTGCGAGCAGGGGCTGTATGGATGGAAAGTGACAGACTGTAAAATCTGTTTTGAATATGGATTGTATTATAGTCCT<br>GTAAGTACCCCCGCAGACTTTCGGCTGCTTTCCCCTATCGTATTGGAGCAGGCTTTAAAAAAAGCAGGGACAGAAC<br>TATTAGAGCCATATCTCCACTTTGAAATTTATGCACCGCAGGAATATCTCTCACGGGCGTATCATGATGCTCCAAGG<br>TATTGTGCAGATATTGTAAGTACTCAGATAAAGAATGACGAGGTCATTCTGAAAGGAGAAATCCCTGCTAGATGTA<br>TTCAAGAATACAGGAACGATTTAACTTATTTCACAAATGGGCAGGGAGTCTGCTTGACAGAGTTAAAAGGATACCA<br>GCCAGCTATTGGTAAATTTATTTGCCAACCCCGCCGCCCGAATAGCCGTATAGATAAGGTTCGGCATATGTTCCACA<br>AGTTAGCTTAA
M20925	*tet*(O)_2	ATGAAAATAATTAACTTAGGCATTCTGGCTCACGTTGACGCAGGAAAGACAACATTAACGGAGAGTTTATTGTATA<br>CCAGTGGTGCAATTGCAGAACCAGGGAGCGTAGATAAAGGCACAACAAGGACAGATACAATGAATTTGGAGCGTC<br>AAAGGGGAATCACTATCCAGACAGCAGTGACATCTTTTCAGTGGGAGGATGTAAAAGTCAACATTATAGATACGCC<br>AGGCCATATGGATTTTTTGGCGGAAGTATACCGTTCTTTATCCGTATTAGACGGAGCAGTATTATTAGTTTCTGCAAA<br>GGATGGCATACAGGCACAGACCCGTATACTGTTTCATGCACTACAGACAATGAAGATTCCGACAATTTTTTTCATCA<br>ATAAAATTGACCAAGAGGGGATTGATTTGCCAATGGTATATCGAGAAATGAAAGCAAAGCTTTCTTCGGAAATTAT<br>AGTGAAGCAAAAGGTTGGGCAGCATCCCCATATAAATGTAACAGACAATGACGATATGGAACAGTGGGATACGGT<br>AATTATGGGAAACGATGAACTATTAGAGAAATATATGTCAGGGAAACCGTTTAAAATGTCAGAACTGGAACAGGA<br>AGAAAACAGGAGATTCCAAAACGGAACGTTATTTCCCGTTTATCACGGAAGCGCTAAAAACAATCTGGGGATTCGG<br>CAGCTTATAGAAGTGATTGCCAGTAAATTTTATTCATCAACGCCTGAAGGTCAATCTGAACTATGCGGGCAGGTTTT<br>TAAGATTGAATATTCAGAGAAAAGGCGGCGTTTTGTTTATGTGCGTATATATAGCGGAACATTGCATTTGAGGGATG<br>TTATTAGAATATCTGAAAAAGAGAAAATAAAAATCACAGAGATGTGTGTTCCGACAAACGGTGAATTATATTCATC<br>CGATACAGCCTGCTCTGGTGATATTGTAATTTTACCAAATGATGTTTTGCAGCTAAACAGTATTTTGGGGAACGAAA<br>TACTGTTGCCGCAGAGAAAATTTATTGAAAATCCTCTCCCTATGCTCCAAACAACGATTGCAGTAAAGAAATCTGAA<br>CAGCGGGAAATATTGCTTGGGGCACTTACAGAAATTTCAGATGGCGACCCTCTTTTAAAATATTATGTGGATACTAC<br>AACGCATGAGATTATACTTTCTTTTTTGGGGAATGTGCAGATGGAAGTCATTTGTGCCATCCTTGAGGAAAAATATC

ATGTGGAGGCAGAAATAAAAGAGCCTACTGTTATATATATGGAAAGACCGCTTAGAAAAGCAGAATATACCATCC<br>ACATAGAAGTCCCGCCAAATCCTTTCTGGGCTTCTGTCGGGTTGTCCATAGAGCCGCTCCCTATTGGAAGCGGAGTG<br>CAGTATGAAAGCAGAGTTTCACTTGGATATTTAAATCAATCGTTCCAAAATGCGGTTATGGAGGGGGTTCTTTATGG<br>CTGCGAGCAGGGGCTGTATGGATGGAAAGTGACAGACTGTAAAATCTGTTTTGAATATGGATTGTATTATAGTCCTG<br>TAAGTACCCCCGCAGACTTTCGGCTGCTTTCCCCTATCGTATTGGAGCAGGCTTTAAAAAAAGCAGGGACAGAACT<br>ATTAGAGCCATATCTCCACTTTGAAATTTATGCACCGCAGGAATATCTCTCACGGGCGTATCATGATGCTCCAAGGT<br>ATTGTGCAGATATTGTAAGTACTCAGATAAAGAATGACGAGGTCATTCTGAAAGGAGAAATCCCTGCTAGATGTAT<br>TCAAGAATACAGGAACGATTTAACTTATTTCACAAATGGGCAGGGAGTCTGCTTGACAGAGTTAAAAGGATACCAG<br>CCAGCTATTGGTAAATTTATTTGCCAACCCCGCCGCCCGAATAGCCGTATAGATAAGGTTCGGCATATGTTCCACAA<br>GTTAGCTTAA

All reference sequences were extracted from resfinder database on webpage https://bitbucket.org/genomicepidemiology/resfinder_db (updated before May 27 2024).

Table S6. The interpretative standards for antibiotic resistance used in this study.

Antibiotics	*C.jejuni*/*C. coli*		
	**Susceptible**	**Intermediate**	**Resistant**
Ciprofloxacin [a]	≤1	2	≥4
Erythromycin [a]	≤8	16	≥32
Doxycycline [a]	≤2	4	≥8
Tetracycline [a]	≤4	8	≥16
Chloramphenicol [b]	≤16	-	≥32
Gentamicin [b]	≤2	-	≥4

[a] From Clinical and Laboratory Standard Institute (CLSI, M45-A3) [b] From EUCAST epidemiological cut-off values (ECOFFs), with wild type as susceptible and non-wild type as resistant. (https://www.eucast.org/mic_and_zone_distributions_and_ecoffs, accessed on 27 May 2024).
